# Nucleolar stress facilitates islet β cell senescence via hijacking the DNA damage response pathways

**DOI:** 10.1016/j.isci.2025.113508

**Published:** 2025-09-04

**Authors:** Yaqi Jiao, Weirong Lu, Xiaohua Wang, Wenxing Sun, Baoying Hu, Jianya Zhao, Jinghao Fang, Ying Lu, Chunhua Wan

**Affiliations:** 1 Department of Nutrition and Food Hygiene, School of Public Health, Nantong University, Nantong, Jiangsu 226019, China; 2 Tongliao Center for Disease Control and Prevention, Inner Mongolia Autonomous Region, Tongliao 028000, China; 3 Department of Endocrinology, The Second Affiliated Hospital of Nantong University, Nantong University, Nantong, Jiangsu 226001, China; 4 Department of Immunology, Medical College, Nantong University, Nantong, Jiangsu 226019, China

**Keywords:** Biochemistry, Cell biology

## Abstract

Senescence is a crucial contributor to pancreatic β cell dysfunction during diabetes progression. Herein, we demonstrated that nucleolar stress, a stress event resulting from disrupted ribosomal RNA (rRNA) synthesis, drives β cell senescence. Senescent β cells exhibited altered nucleolar morphology and redistribution of the nucleolar protein nucleophosmin (NPM) *in vivo*. Exposure to nucleolar stress inducers CX-5461 and actinomycin D (ActD) resulted in senescence-associated β-gal staining (SA-β-gal) activity in cultured β cells. This was accompanied by upregulation of senescence markers p53, p21, and p16 and a senescence-associated secretory phenotype (SASP). Notably, nucleolar stress also induced γ-H2AX foci formation and Ataxia telangiectasia mutated (ATM) activation independently of DNA double-strand breaks (DSBs). Pharmacological inhibition of ATM with KU60019 strongly attenuated nucleolar stress-induced β cell senescence. Collectively, these findings identify nucleolar stress as a key upstream event in β cell senescence and highlight the γ-H2AX-ATM axis as a critical mediator of this process.

## Highlights

• Metabolic stress induces nucleolar disruption and stress in senescent β cells
• Chemical induction of nucleolar stress drives β cell senescence
• Nucleolar stress activates the γ-H2AX/ATM pathway independently of DSBs
• ATM inhibition prevents nucleolar stress-induced β-cell senescence

## Graphical abstract

**Figure:**
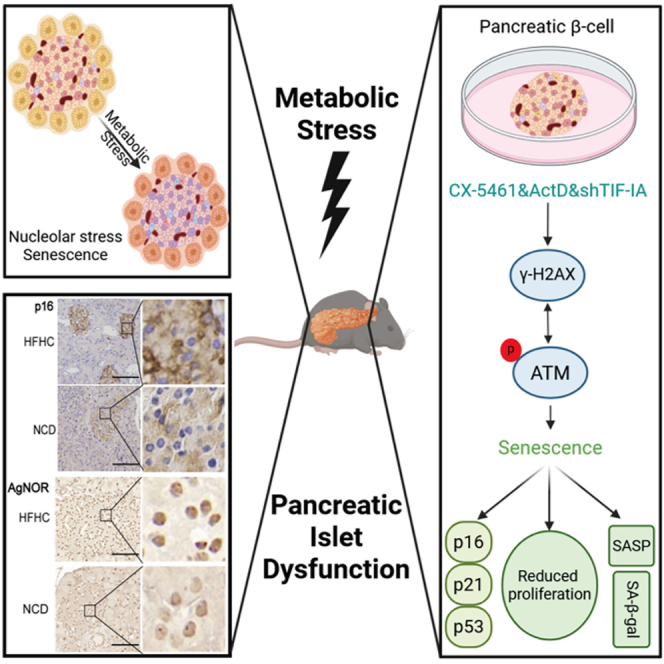

## Introduction

Diabetes mellitus (DM) is a chronic metabolic syndrome characterized by persistent hyperglycemia and insufficient insulin secretion.^1^^,^^2^ It mainly comprises type 1 and type 2 DM, both involving progressive dysfunction and loss of pancreatic β cells. Following exposure to unfavorable stimuli, such as high glucose, pro-inflammatory cytokines, and saturated fatty acid, pancreatic β cells undergo various pathological alterations, including apoptosis, senescence, and autophagy. Studies in the past two decades have revealed that cellular senescence plays an integral role in β cell dysfunction. In a high-fat diet-induced diabetic model, Sone and Kagawa reported that β cell senescence is associated with the impairment in β cell proliferation and insulin secretion.^3^ Paradoxically, other studies have reported that β cell senescence may enhance β cell maturation and insulin secretion.^4^ However, the prevailing view suggests that senescence leads to β cell functional decline and metabolic deterioration, whereas the removal of senescent β cells improves glucose metabolism and type 2 DM progression.^5^ Likewise, β cell senescence plays both detrimental and beneficial roles in type 1 DM development, depending on the disease stages and molecular signature.^6^^,^^7^^,^^8^ These studies underscore the complex and context-dependent roles of β cell senescence in diabetes pathogenesis.

β cell senescence is characterized by common senescence features, including increased senescence-associated β-gal staining (SA-β-gal) activity, elevated expression of senescent markers p16 and p21, and a senescence-associated secretory phenotype (SASP).^9^^,^^10^ Emerging evidence indicated that β cell senescence can be initiated by a variety of stress stimuli, for instance, unfolded protein response stress, metabolic stress, and DNA damage.^7^^,^^11^^,^^12^^,^^13^ Notably, these diverse stress stimuli often converge on common molecular pathways, such as oxidative stress, γ-H2AX foci formation, and p53 activation, to initiate senescence program.^10^^,^^11^^,^^13^^,^^14^ These findings align with the free radical hypothesis of aging, which posits that persistent reactive oxygen species (ROS)-induced DNA damage responses (DDR) are key drivers of cellular senescence.^15^ While metabolic stress has been implicated in promoting β cell senescence through DDR activation, the underlying mechanisms remain incompletely understood. For instance, metabolic stress-induced DNA double-strand breaks (DSBs), owing to their highly cytotoxic nature, are typically associated with β cell apoptosis rather than senescence.^16^^,^^17^ Interestingly, emerging studies have also highlighted the role of novel stress-sensing mechanisms, such as nucleolar stress, in linking various stress signals to DDR pathways, particularly through the activation of p53 signaling.^18^

Nucleolar stress is a cellular stress event caused by disrupted ribosomal RNA (rRNA) transcription and nucleolar morphology. It can be triggered by a range of stress stimuli, including nutrient deprivation, hypoxia, DNA damage, and oxidative and thermal stresses.^18^ Notably, nucleolar stress can facilitate assorted signaling pathways that influence cell fate and disease progression.^19^ For instance, the nucleolus has been well recognized as a sensor of DNA damage and may trigger the DDR.^20^ Nucleolar stress can activate critical DDR-related signaling pathways, including p53, Ataxia telangiectasia mutated and Rad3-related (ATR)-checkpoint kinase 1, and Ataxia telangiectasia mutated (ATM).^21^ Through these mechanisms, it plays a role in regulating cell apoptosis, premature senescence, and autophagy.^19^ Although rRNA synthesis is tightly regulated by various metabolic signaling pathways, such as AMPK, mTOR, and Sirt1, the specific role of nucleolar stress in the development of metabolic diseases remains largely unexplored.^19^

In the present study, we investigated the involvement of nucleolar stress in pancreatic β cell physiology and identified it as a crucial driver of β cell senescence. Molecular markers of nucleolar stress were specifically detected in senescent β cells from high-fat high-cholesterol (HFHC) diet-induced diabetic mice. Pharmacological inhibition of RNA polymerase I (Pol I) with CX-5461 or actinomycin D (ActD) induced β cell senescence in a concentration-dependent manner. Nucleolar stress may lead to pseudo-DDR characterized by γ-H2AX foci formation and ATM activation. Pharmaceutical blockade of ATM markedly attenuated senescent phenotype of β cells. These findings underscore nucleolar stress as a crucial initiator of β cell senescence, highlighting intervening approaches targeting nucleolar stress and its downstream events as novel therapeutic strategies against β cell mass decline and diabetic progression.

## Results

### The presence of nucleolar aberration during metabolic stress-induced ontogeny of senescence

**Figure 1:**
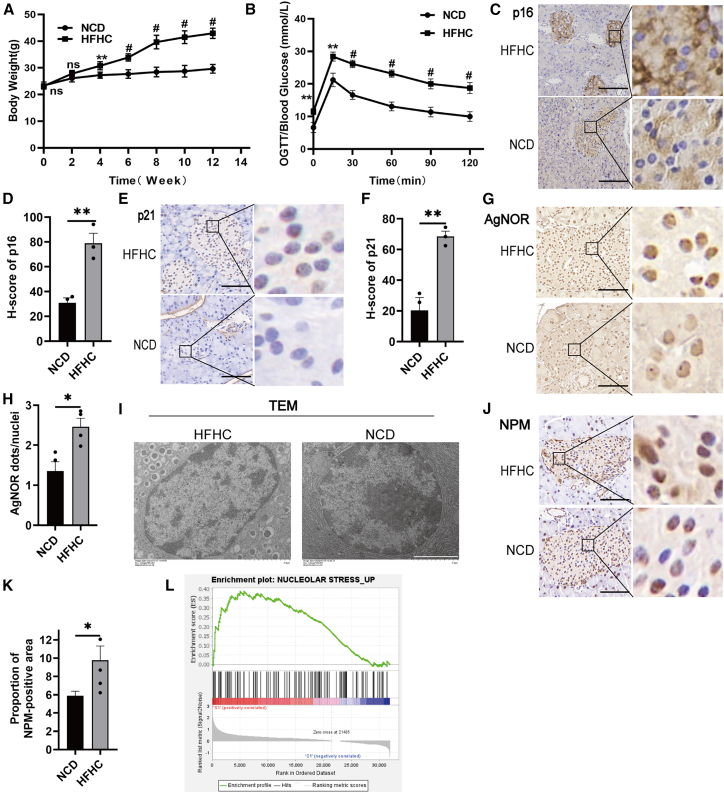
The occurrence of nucleolar stress markers in metabolic stress-induced senescence of pancreatic islet cells (A) Changes in body weight of mice fed with a normal diet and a 42% fat and 0.2% cholesterol diet for 12 weeks. Data are mean ± SEM; *n* = 6 independent biological replicates. Two-way ANOVA with Sidak adjustment: *p*∗∗ < 0.01, *p*^#^ < 0.001. (B) Blood glucose levels measured by an oral glucose tolerance test (OGTT) on mice after 12 weeks of feeding. Data are mean ± SEM; *n* = 3 independent biological replicates. Two-way ANOVA with Sidak adjustment: *p*∗∗ < 0.01, *p*^#^ < 0.001. (C–F) Immunohistochemistry of p16 (C) and p21 (E) in islets of normal and HFHC diet mice (scale bars, 20 μm). H scores for immunohistochemistry of p16 (D) and p21 (F). Data are mean ± SEM; *n* = 3 independent biological replicates. Student’s t test: *p*∗∗ < 0.01. (G) Nucleolar silver staining was carried out on islets of normal and HFHC diet mice (scale bars, 20 μm). (H) Statistical analysis of nucleolar quantity. Data are mean ± SEM; *n* = 4. Student’s t test: *p∗* < 0.05. (I) TEM of pancreatic islets from normal and HFHC mice (scale bars, 2 μm). (J) Immunohistochemistry of NPM in mouse islets (scale bars, 20 μm). (K) Quantitative analysis of NPM immunohistochemistry. Data are mean ± SEM; *n* = 4. Student’s t test: *p*∗ < 0.05. (L) GSEA analysis of the nucleolar stress signaling pathway in senescent islet β cells. The nucleolar stress gene set was derived from the gene expression dataset GEO (GSE239319) and utilized for GSEA analysis on the expression dataset GSE121539. See also Figure S1.

We first assessed whether metabolic-stress-induced β cell malfunction and senescence involves nucleolar aberration. Following a 12-week period on a HFHC, the body weight of the mice exhibited a notable increase. Additionally, overt hyperglycemia was evident in the islet tissues, accompanied by distinct characteristics of cellular senescence (Figures 1A and 1B). Immunohistochemical analysis revealed marked increases in the expression of senescence markers p16 and p21 in the pancreatic islets of HFHC mice, compared with tissues of chow diet-fed mice (Figures 1C–1F).

To examine the alterations in nucleolar morphology, we employed transmission electron microscope (TEM) and argyrophilic nucleolar organizer region to assess the number and area of the nucleoli between the two groups. Islet cells from HFHC mice exhibited a significantly increased number of nucleoli per cell, suggesting that the nucleoli of islet cells may be more fragmented following metabolic stress (Figures 1G–1I). Accordingly, significantly enhanced diffusion of nucleophosmin (NPM), a hallmark phenomenon of nucleolar stress, was observed from the nucleolus to the nucleoplasm in the islet cells of HFHC-fed mice (Figures 1J and 1K). This phenomenon was recapitulated in cultured β cells treated with established stress inducers (palmitic acid and H_2_O_2_; Figures S1A and S1B). Notably, gene set enrichment analysis (GSEA) revealed a notable upregulation of gene set involved in nucleolar stress in senescent islet cells in mice (Figure 1L). These findings implicate that nucleolar stress may play a facilitating role in the process of pancreatic islet senescence.

### Exposure to nucleolar stress inducers CX-5461 and ActD triggers senescent phenotype in pancreatic β cells

To decipher the involvement of nucleolar stress in the induction of β cell senescence, we examined the senescent phenotypes in two established pancreatic β cell lines: mouse β-TC-6 and rat INS-1 cells. We utilized two established chemical inhibitors of RNA Pol I, CX-5461 and ActD, to induce nucleolar stress. Both compounds selectively target ribosomal DNA and inhibit Pol I-mediated rRNA transcription at appropriate concentrations.^22^^,^^23^ Consistent with their known mechanisms, treatment with these compounds effectively induced nucleolar stress, as demonstrated by prominent nucleoplasmic translocation of NPM (Figure S1C) and significant reduction in all major rRNA species (Figure S2).

**Figure 2:**
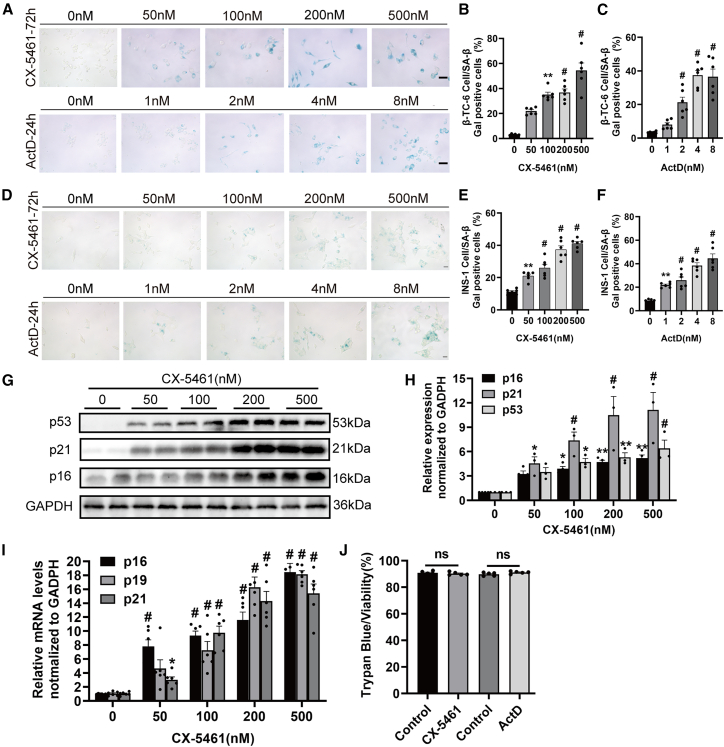
CX-5461 and ActD induce senescence phenotypes in pancreatic β cells (A–F) SA-β-gal staining of β-TC-6 (A) and INS-1 (D) cells following exposure to CX-5461 or ActD for 72 or 24 h, respectively (scale bars, 20 μm). SA-β-gal staining-positive cells of β-TC-6 (B and C) and INS-1 (E and F) were quantitatively analyzed. Data are mean ± SEM; *n* = 6, technical replicates. Dunnett’s HSD test compared with the control: *p*∗∗ < 0.01, *p*^#^ < 0.001. (G) Western blot analysis of p16, p19, and p21 proteins in β-TC-6 cells following CX-5461 treatment for 72 h. The duplicate samples shown represent independent biological replicates. (H) Quantitative analysis of the levels of p16, p19, and p21 proteins. Data are mean ± SEM; *n* = 3 independent biological replicates. Dunnett’s HSD test compared with the control: *p*∗∗ < 0.01, *p*^#^ < 0.001. (I) Real-time qPCR analysis of the mRNA levels of p16, p19, and p21 in CX-5461-exposed β-TC-6 cells. Data are mean ± SEM; *n* = 6. Dunnett’s HSD test compared with the control: *p*^ns^ > 0.05, *p*∗∗ < 0.01, *p*^#^ < 0.001. (J) Cell viability assessed by trypan blue staining assay in β-TC-6 cells after exposure to 500 nM CX-5461 and 8 nM ActD. Data are mean ± SEM; *n* = 5. Student’s t test: *p*^ns^ > 0.05.

**Figure 3:**
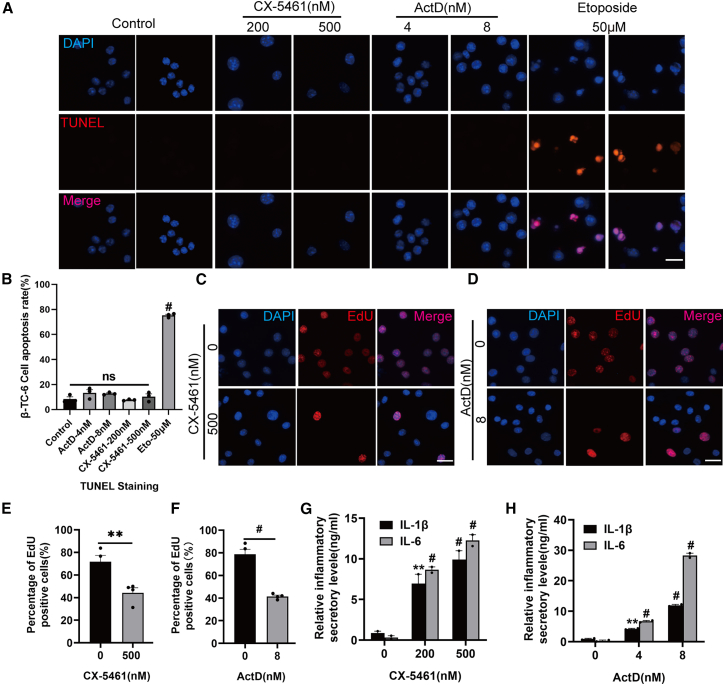
Exposure to CX-5461 and ActD induces proliferation impairment and SASP (A) TUNEL apoptotic assay of cells treated with CX-5461 or ActD. Etoposide-treated groups serve as the positive control (scale bars, 20 μm). (B) Quantitative analysis of TUNEL-positive cells in the indicated groups. Data are mean ± SEM; *n* = 3, technical replicates. Dunnett’s HSD test compared with the control: *p*^ns^ > 0.05, *p*^#^ < 0.001. (C–F) The proliferation of β-TC-6 cells exposed to 500 nM CX-5461 (C) and 8 nM ActD (D) was evaluated using the EdU incorporation assay (scale bars, 20 μm). Quantitative analysis of the EdU-positive cells following CX-5461 (E) and ActD (F) exposure. Data are mean ± SEM; *n* = 4. Student’s t test: *p*∗∗ < 0.01. (G and H) ELISA analysis of secreted levels of IL-1β and IL-6 in β-TC-6 cells after exposure to CX-5461 (G) and ActD (H). Data are mean ± SEM; *n* = 2 independent biological replicates. Dunnett’s HSD test compared with the control: *p*∗∗ < 0.01, *p*^#^ < 0.001.

SA-β-gal staining revealed that both nucleolar stress inducers significantly drive senescent phenotype of β cell, in a dose-dependent manner (Figures 2A–2F). Importantly, with these dose ranges, we did not observe any evidence of β cell apoptosis, as assessed by TUNEL staining (Figures 3A and 3B). Furthermore, we examined the levels of established senescence markers, including p16, p19, and p21, in β-TC-6 cells. Notably, we observed a significant increase in the levels of these proteins following CX-5461 exposure (Figures 2G and 2H). Similar results were obtained at the mRNA levels (Figure 2I).

One of the defining characteristics of cellular senescence is the irreversible cessation of cell proliferation. The EdU incorporation assay revealed that nucleolar stress stimulants resulted in a notable decline in the number of proliferating cells (Figures 3D–3F), without the involvement of cell death (Figure 2J). It is noteworthy that the levels of interleukin (IL)-1β and IL-6, hallmark components of the SASP, were markedly elevated in both cell types (Figures 3G and 3H). This observation indicates the apparent onset of cellular senescence and further supports the hypothesis that nucleolar stress induction can trigger senescent phenotypes in established β cells.

### Genetic inhibition of rRNA synthesis via TIF-IA depletion induces β cell senescence

**Figure 4:**
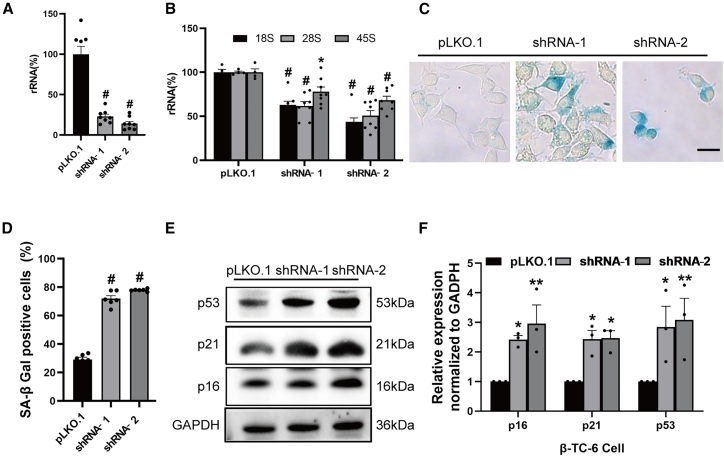
TIF-IA knockdown impairs rRNA synthesis and induces β cell senescence (A) Quantitative analysis of TIF-IA expression in β-TC-6 cells following TIF-IA depletion. Data are mean ± SEM; *n* = 8. Dunnett’s HSD test compared with the control: *p*^#^ < 0.001. (B) Real-time qPCR analysis of 18S, 28S, and 45S rRNA expression levels in TIF-IA-depleted β-TC-6 cells. Data are mean ± SEM; *n* = 8. Dunnett’s HSD test compared with the control: *p*∗ < 0.05, *p*^#^ < 0.001. (C) Assessment of cellular senescence phenotype via SA-β-gal staining (scale bars, 20 μm). (D) Quantification of SA-β-gal-positive cell proportion. Data are mean ± SEM; *n* = 6. Dunnett’s HSD test compared with the control: *p*^#^ < 0.001. (E) Western blot analysis of senescence-associated protein expression including p16, p21, and p53 following TIF-IA depletion. (F) Quantitative analysis of senescence-associated protein expression. Data are mean ± SEM and analyzed using one-way ANOVA. Data are mean ± SEM; *n* = 3 independent biological replicates. Dunnett’s HSD test compared with the control: *p*∗∗ < 0.01, *p*^#^ < 0.001. See also Figure S2.

To clarify the specific role of nucleolar stress in β cell senescence, we investigated whether genetically induced nucleolar stress also triggers β cell senescence. rRNA transcription by the RNA Pol I machinery requires multiple essential transcriptional co-factors, including transcription initiation factor IA (TIF-IA/Rrn3) and upstream binding factor. Disruption of their expression can effectively impair rRNA synthesis and induce nucleolar stress.^19^ Accordingly, we generated lentiviral short hairpin RNA constructs targeting mouse TIF-IA and transduced them into β-TC-6 cells. Quantitative real-time PCR (real-time qPCR) analysis confirmed a significant reduction in TIF-IA mRNA levels following knockdown (Figure 4A).

To evaluate the extent of nucleolar dysfunction, we analyzed the expression of key rRNA transcripts, including 18S, 28S, and their 45S precursor. TIF-IA-depleted cells exhibited substantial reductions in all three rRNA species (Figure 4B), mirroring the effects observed with chemical nucleolar stress inducers (Figure S2). To establish a causal relationship between nucleolar stress and senescence, multidimensional senescent phenotypes were assessed in TIF-IA-depleted cells. Notably, TIF-IA depletion resulted in robust SA-β-gal activity (Figures 4C and 4D) and elevated expression of senescent markers, including p16, p21, and p53 (Figures 4E and 4F). These results demonstrate that genetic inhibition of rRNA transcription via TIF-IA depletion induces nucleolar stress and β cell senescence.

### Nucleolar stress-induced β cell senescence involves γ-H2AX foci formation

**Figure 5:**
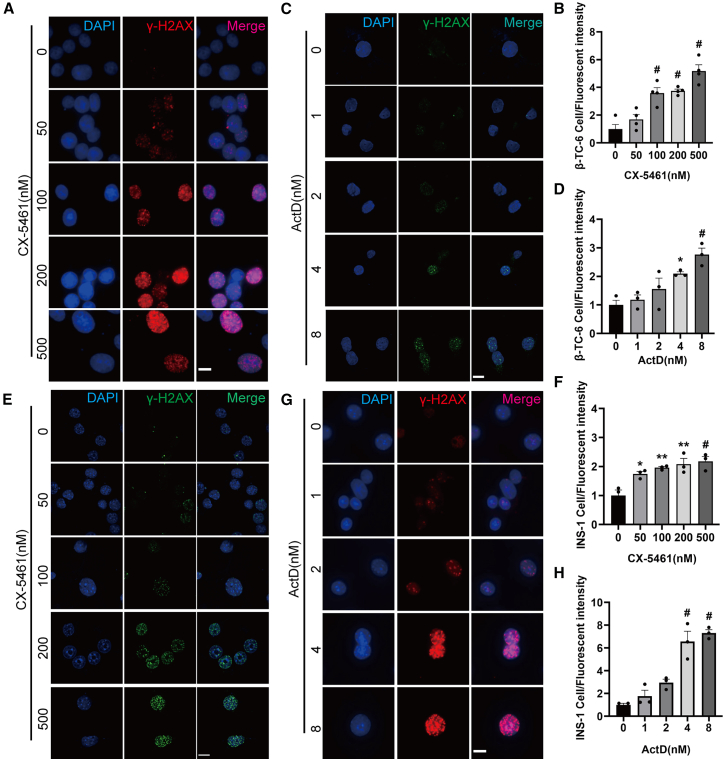
Exposure to CX-5461 and ActD induces the formation of γ-H2AX foci in β cells (A–D) β-TC-6 cells were exposed to the indicated concentrations of CX-5461 (A) for 72 h or ActD (C) for 24 h (scale bars, 10 μm) and subjected to immunofluorescence analysis of γ-H2AX foci formation. Quantitative analysis of the intensities of γ-H2AX foci in β-TC-6 cells following exposure to CX-5461 (B) and ActD (D). Data are mean ± SEM; *n* = 3 independent biological replicates. Dunnett’s HSD test compared with the control: *p*∗∗ < 0.01, *p*^#^ < 0.001. (E–H) Immunofluorescence analysis of INS-1 cells following exposure to CX-5461 (E) for 72 h or ActD (G) for 24 h (scale bars, 10 μm). Quantitative analysis of the intensities of γ-H2AX foci following CX-5461 (F) or ActD (H) exposure. Data are mean ± SEM; *n* = 3. Dunnett’s HSD test compared with the control: *p*∗∗ < 0.01, *p*^#^ < 0.001.

To further elucidate the molecular mechanism by which nucleolar stress induces β cell senescence, we examined the molecular alterations in β cells following CX-5461 and ActD exposure. Because nucleolar stress has been closely associated with the activation of DDR pathways, we investigated whether nucleolar stress may result in molecular alterations in DDR. Specifically, we analyzed the formation of DNA damage foci using an anti-γ-H2AX antibody. As anticipated, CX-5461 exposure caused apparent formation of γ-H2AX foci in β cells in a dose-dependent manner, especially at CX-5461 doses of 100–500 nM range (Figures 5A and 5B). Likewise, ActD treatment produced a comparable increase in γ-H2AX foci at a dose range of 0–8 nM (Figures 5C and 5D). The findings were validated in INS-1 cells (Figures 5E–5H). These findings implicate the activation of the DDR pathway as a contributing mechanism in the nucleolar stress-induced senescent phenotype of β cells.

### Nucleolar stress initiates ATM activation

Previous studies have suggested that senescence-inducing agents such as ultraviolet irradiation, γ-radiation, or H_2_O_2_ may induce DNA oxidative damage and γ-H2AX foci to drive the senescent phenotype.^24^^,^^25^^,^^26^ These events may trigger the activation of downstream DDR pathways, leading to senescent induction in various cell types.^27^^,^^28^

**Figure 6:**
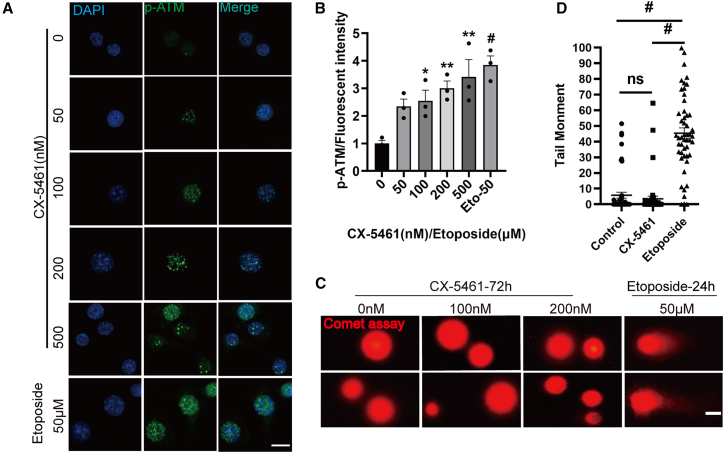
Nucleolar stress induces γ-H2AX foci formation and ATM activation without the occurrence of DSBs in β cells (A) Immunofluorescence analysis of phosphorylated ATM (p-ATM) in β-TC-6 cells exposed to CX-5461 for 72 h (scale bars, 10 μm). (B) Quantitative analysis of p-ATM fluorescence intensity. Data are mean ± SEM; *n* = 3 independent biological replicates. Dunnett’s HSD test compared with the control: *p*∗ < 0.05, *p*∗∗ < 0.01, *p*^#^ < 0.001. (C) Comet assay was conducted in β-TC-6 cells exposed to CX-5461 for 72 h, with 50 μM etoposide serving as a positive control (scale bars, 10 μm). (D) Statistical analysis of the comet tail length (50 cells per group). Data are mean ± SEM; *n* = 50, technical replicates. Student’s t test: *p*^ns^ > 0.05, *p*^#^ < 0.001. See also Figure S3.

To assess the involvement of DDR pathways in these processes, we investigated the activation of ATM and ATR pathways using immunofluorescence assay to detect their phosphorylated forms. We revealed significant phosphorylated ATM foci following CX-5461 exposure in a concentration-dependent manner, while phosphorylated ATR foci was minimally observed under the same conditions (Figures 6A, 6B, and S3). This finding provides further evidence that nucleolar stress promotes the senescence of β cells via activating the ATM pathway.

### Nucleolar stress induces β cell senescence via pseudo-DNA damage and ATM activation

Serving as critical markers of DNA damage, γ-H2AX foci are crucially implicated in sensing DNA DSBs. However, accumulating evidence indicates the formation of γ-H2AX foci in the absence of DSBs in senescent cells.^29^^,^^30^ To evaluate whether nucleolar stress-induced γ-H2AX foci reflect DSBs, we performed comet assay to investigate the occurrence of DSBs following nucleolar stress. DNA topoisomerase II inhibitor etoposide was used as a positive control. While etoposide treatment resulted in clear DNA comet tails indicative of DSBs, CX-5461 exposure did not produce similar evidence of DNA fragmentation (Figures 6C and 6D). These findings suggest that nucleolar stress-induced γ-H2AX foci are uniquely associated with cellular senescence without actual DSBs.

**Figure 7:**
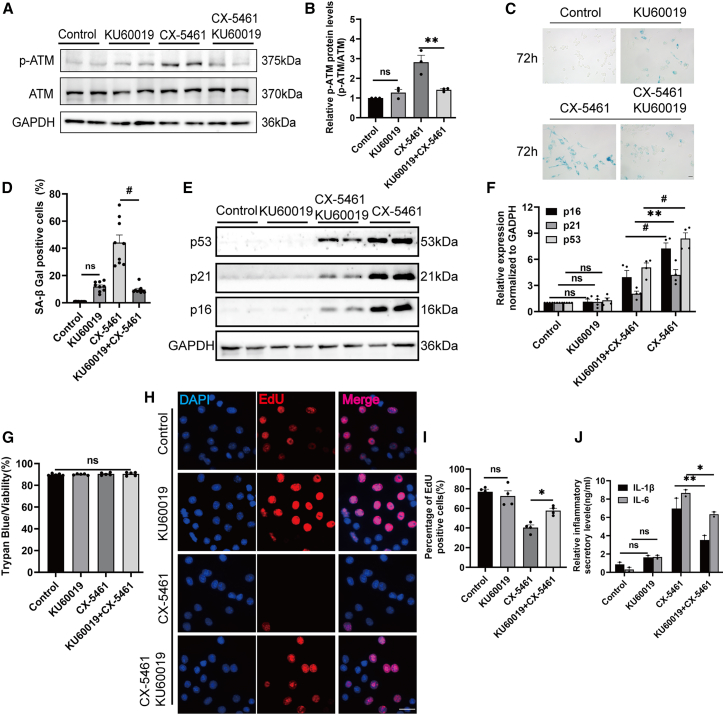
Inhibition of ATM activation can effectively suppress the senescence of β-TC-6 cells and the secretion of inflammatory factors induced by nucleolar stress (A) Western blot analysis of ATM and p-ATM in β-TC-6 cells with or without exposure to 200 nM CX-5461 and 3 μM KU60019 for 72 h. The duplicate samples shown represent independent biological replicates. (B) Quantitative analysis of p-ATM/ATM ratios in the indicated groups. Data are mean ± SEM; *n* = 3 independent biological replicates. Two-way ANOVA with Sidak adjustment: *p*^ns^ > 0.05, *p*^∗∗^ < 0.01. (C) SA-β-gal analysis of β-TC-6 following the indicated treatments (scale bars, 10 μm). (D) Quantitative analysis of SA-β-gal staining-positive cells. Data are mean ± SEM; *n* = 9, technical replicates. Two-way ANOVA with Sidak adjustment: *p*^ns^ > 0.05, *p*^#^ < 0.001. (E) Western blot analysis of p16, p19, and p21 proteins in β-TC-6 cells following the indicated treatments. The duplicate samples shown represent independent biological replicates. (F) Quantitative analysis of the expression of p16, p19, and p21 proteins. Data are mean ± SEM; *n* = 4 independent biological replicates. Two-way ANOVA with Sidak adjustment: *p*^ns^ > 0.05, *p*^∗∗^ < 0.01, *p*^#^ < 0.001. (G) The proportion of viable cells was detected by the trypan blue assay. Data are mean ± SEM; *n* = 5. Student’s t test: *p*^ns^ > 0.05. (H) EdU incorporation assay of β-TC-6 cell proliferation with or without CX-5461 and KU60019 treatments (scale bars, 20 μm). (I) Quantitative analysis of the EdU-positive cells. Data are mean ± SEM; *n* = 4, technical replicates. Two-way ANOVA with Sidak adjustment: *p*^ns^ > 0.05, *p*^∗^ < 0.05. (J) The levels of secreted IL-1β and IL-6 in the indicated groups were assessed using ELISA assay. 0 was used as a positive control. Data are mean ± SEM; *n* = 2 independent biological replicates. Two-way ANOVA test with Sidak adjustment: *p*^ns^ > 0.05, *p*^∗^ < 0.05, *p*^∗∗^ < 0.01.

To investigate the causal relationship between DNA damage pathways and β cell senescence, we employed the ATM inhibitor KU60019 to inhibit ATM activation. Co-treatment with KU60019 strongly attenuated ATM phosphorylation induced by CX-5461 exposure in β cells (Figures 7A and 7B). Furthermore, treatment with KU60019 blocked CX-5461-induced SA-β-gal staining in β cells (Figures 7C and 7D). Importantly, western blot results indicated that co-incubation with KU60019 substantially impaired the expression of senescence marker proteins, including p53, p21, and p16, in CX-5461-exposed β cells (Figures 7E and 7F).

Consistent with these findings, EdU incorporation assay demonstrated that the percentage of proliferating cells was restored upon co-incubation with KU60019, compared to cells treated with CX-5461 alone (Figures 7H and 7I). This recovery occurred without detectable reductions in cell viability, as measured by trypan blue assay (Figure 7G). Furthermore, the elevated levels of IL-1β and IL-6 in the cells were attenuated following KU60019 treatment (Figure 7J). These results highlight the critical role of ATM activation in the nucleolar stress-induced β cell senescence.

### Nucleolar stress induces γ-H2AX foci formation and upregulates p53 and p16 in primary islet cells

**Figure 8:**
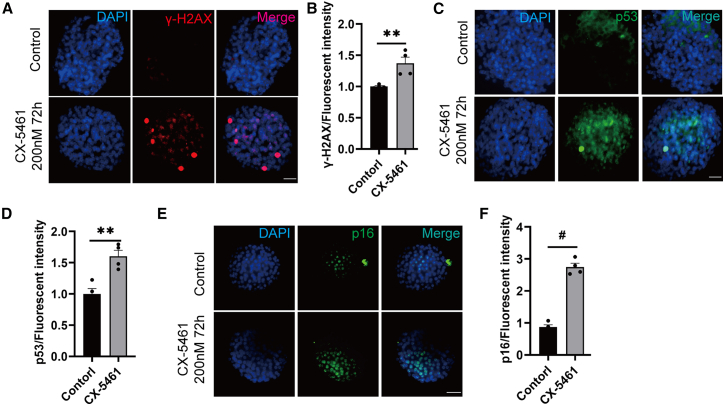
CX-5461 induces DNA damage markers and senescence proteins in primary pancreatic islets (A) Immunofluorescence staining of γ-H2AX in mouse primary islets treated with 200 nM CX-5461 for 72 h (scale bars, 20 μm). (B) Quantification of γ-H2AX fluorescence intensity. Data are mean ± SEM; *n* = 4, technical replicates. Student’s t test: *p*^∗∗^ < 0.01. (C–F) Immunofluorescence of p53 (C) and p16 (E) in CX-5461-treated islets (scale bars, 20 μm). Quantitative analysis of p16 (D) and p53 (F) fluorescence intensities. Data are mean ± SEM; *n* = 4. Student’s t test: *p*^∗∗^ < 0.01, *p*^#^ < 0.001.

To validate the effect of nucleolar stress in inducing the islet β cell senescent program, we isolated primary islets from C57/B6 mice and exposed them to CX-5461 for 72 h. As expected, CX-5461 exposure led to a marked increase in γ-H2AX foci formation, compared to the vehicle control (Figures 8A and 8B). Moreover, the examination of senescence markers revealed significantly elevated expression of both p53 and p16 in CX-5461-treated islets (Figures 8C–8F). Taken together, these data indicate that nucleolar stress can elicit a pseudo-DDR and induce p53 and p16 activation in primary islet cells.

## Discussion

Pancreatic β cell failure is a prerequisite for diabetic onset and progression. Recent investigations have highlighted an integral role of cellular senescence in β cell dysfunction and loss during diabetic progression. However, the critical stress events leading to β cell senescence remain largely obscure. Our present study showed that nucleolar stress may initiate a pseudo-DDR characterized by γ-H2AX foci formation in the absence of actual DSBs. This response leads to ATM activation, which promotes β cell senescence. Notably, pharmacological inhibition of ATM significantly attenuated nucleolar stress-induced β cell senescence. Taken together, our findings suggest that nucleolar stress may serve as a general stress event to facilitate pancreatic β cell senescence during diabetic progression.

Nucleolar stress has been well recognized as an important stress sensor and signal hub when cells encounter a wide range of stress stimuli.^31^ Notably, the initiation of nucleolar stress has been shown to facilitate signaling events leading to cell death, autophagy, differentiation, and senescence. Nucleolar stress triggered by genetic ablation of TIF-IA, an essential transcription factor for rRNA synthesis, results in smooth muscle cell senescence and vascular degeneration via the ATM/ATR-p53 axis.^32^ Likewise, depletion of other nucleolar proteins, such as NOL12 and Sirt7, triggers an apparent senescent phenotype.^33^^,^^34^ In line with these data, nucleolar stress caused by chemical exposure, such as cyclopentenyl cytosine and doxorubicin, also strongly facilitates the senescent program in assorted cell types.^35^^,^^36^ In line with these findings, we showed that nucleolar stress can effectively trigger a senescent phenotype in pancreatic β cells. Although oral administration of CX-5461 failed to induce β cell nucleolar stress and senescence in HFHC diet-fed mice (data not shown), our *in vitro* studies clearly demonstrated that both genetic and chemical nucleolar stress inducers robustly triggered β cell senescence. This was evidenced by increased SA-β-gal activity, elevated expression of senescence markers (p16, p21, and p53), and secretion of SASP factors. These findings highlight nucleolar stress as a potent initiator of β cell senescence. Because nucleolar stress may serve as a stress event downstream of various unfavorable stimuli, such as ROS and metabolic stress, we propose that nucleolar stress may play an integral role in the metabolic stress-facilitated senescence program in pancreatic β cells during diabetic progression.

Studies in the past decades have well characterized that the nucleolus is a sensor of various stress signals, particularly in the surveillance of genomic stability. DNA damage may disrupt the transcription of rRNA, leading to nucleolar architecture alteration and the initiation of nucleolar stress, which in turn is critical for monitoring genome integrity. Some studies have revealed that nucleolar stress is critical for the activation of multiple DDR pathways under genotoxic stress, such as p53, PCNA, ATM, and RAD51,^21^^,^^37^ Notably, nucleolar stress induced by chemical or genetic inhibition of rRNA transcription may activate these DNA damage-associated pathways, even in the absence of DSBs or other forms of DNA damage.^38^^,^^39^

As a key kinase involved in DDR, ATM has been well documented to play a crucial role in senescent regulation. ATM may drive the senescent program via assorted substrate proteins, such as p53, p53-binding protein 1, γ-H2AX, checkpoint kinase-2, and nibrin/NBN.^40^ Consistently, we demonstrated a key role of ATM in nucleolar stress-induced β cell senescence and found that the inhibition of ATM abrogated the β cell senescent phenotype following nucleolar stress. However, the mechanisms underlying ATM activation during cell senescence remains elusive. Although ATM activation has been classically linked to DNA damages, particularly DSBs, studies showed that it can also be activated independently of DSBs, for example, by oxidative stress or the Pol I inhibitor CX-5461.^41^^,^^42^ Notably, ATM and its upstream regulator Nijmegen breakage syndrome 1 can accumulate in nucleolar caps, implicating nucleolar-localized activation.^43^^,^^44^ Our findings align with these reports, demonstrating that nucleolar stress activates ATM to promote β cell senescence and highlighting a non-canonical activation pathway. Moreover, the ATR pathway remained inactive during this process, implying mechanistic specificity. Further studies are needed to fully elucidate the underlying mechanisms.

In summary, our present results indicated that nucleolar stress facilitates β cell senescence via pseudo-DNA damage and ATM activation. Nucleolar stress is observed in metabolic stress-induced senescent β cells, suggesting a broad involvement of nucleolar stress in β cell deterioration during diabetic progression. Blockade of ATM strongly attenuated senescent phenotypes induced by nucleolar stress, highlighting targeting ATM as a feasible approach for preventing excessive β cell senescence and loss under metabolic stress. These data together highlight the therapeutic potential of blocking nucleolar stress-mediated ATM signaling to preserve β cell function and prevent diabetes progression.

### Limitations of the study

This study has several limitations. First, the research scope is confined to *in vitro* cell models and specific *in vivo* metabolic stress conditions, without validation across distinct diabetic subtypes or clinical specimens, potentially limiting translational relevance. Second, while γ-H2AX foci formation was observed following nucleolar stress, the precise molecular mechanisms underlying this process remain unresolved, and we cannot rule out potential interactions with other stress pathways (e.g., oxidative stress or endoplasmic reticulum stress). Third, the activation of ATM by nucleolar stress was characterized phenotypically and via signaling alterations, but the upstream regulators and downstream effectors linking ATM to senescence remain unresolved, leaving critical mechanistic gaps for future investigation. Finally, the study was limited by the investigations on male-derived islets. Sex and sex hormones are well-established modulators of islet cell function, including β cell aging and senescence, and prior studies have shown that males are generally more susceptible to β cell dysfunction and failure.^45^^,^^46^^,^^47^ While our findings demonstrated a strong association between nucleolar stress and pancreatic β cell senescence in male mice and primary islet cultures, it remains unclear to what extent female sex may confer protection, warranting further investigation.

## Resource availability

### Lead contact

Further information and requests for resources and reagents should be directed to and will be fulfilled by the lead contact, Chunhua Wan (chwan@ntu.edu.cn).

### Materials availability

Plasmids generated in this study can be requested from the lead contact. A completed materials transfer agreement and/or payment may be required if the request involves potential commercial applications.

### Data and code availability

• All data reported in this article will be shared by the lead contact upon request.
• This article does not report original code.
• Any additional information required to reanalyze the data reported in this article is available from the lead contact upon request.

## Acknowledgments

This work was supported by grants from the 10.13039/501100001809National Natural Science Foundation of China (82273206 and 81972279), 10.13039/501100018557Nantong Science and Technology Project (MS2022026, JC2023101, MS2024057, and JC12022026), the innovation and entrepreneurship training program of 10.13039/501100005054Nantong University (202310304134Y), the postgraduate research and practice innovation program of 10.13039/501100002949Jiangsu Province (KYCX25_3823), and the open fund of 10.13039/501100005054Nantong University large instruments and equipment (KFJN2445).

## Author contributions

Y.J., W.L., X.W., and J.F. performed the experiments and analyzed the data. C.W., W.S., J.Z., and Y.L. provided key technical guidance and resources. C.W., Y.L., and B.H. designed the study. C.W. and Y.J. wrote the article. All authors discussed the results and commented on the article.

## Declaration of interests

The authors declare no competing interests.

## STAR★Methods

### Key resources table

**Table:** 

REAGENT or RESOURCE	SOURCE	IDENTIFIER
**Antibodies**		

Rabbit monoclonal anti-CDKN2A/p16INK4a	Abcam	Cat# ab211542; RRID: AB_2891084
Rabbit monoclonal anti-p21	Abcam	Cat# ab188224; RRID: AB_2734729
Mouse monoclonal anti-p53 (1C12)	Cell Signaling Technology	Cat# 2524; RRID: AB_331743
Rabbit monoclonal anti-ATM	Sigma-Aldrich	Cat# ZRB1376; RRID: AB_3698743
*p*-Atm Antibody (10H11.E12)	Santa Cruz Biotechnology	Cat# sc-47739; RRID: AB_781524
Rabbit monoclonal anti-phospho-histone H2A.X (Ser139)	Beyotime	Cat# AF1201; RRID: AB_2920717
Rabbit polyclonal anti-GAPDH	Santa Cruz Biotechnology	Cat# sc-25778; RRID: AB_10167668
Goat Polyclonal anti-mouse IgG-HRP	Santa Cruz Biotechnology	Cat# sc-2005; RRID: AB_631736
Goat Polyclonal anti-rabbit IgG-HRP	Santa Cruz Biotechnology	Cat# sc-2004; RRID: AB_631746
Rabbit monoclonal anti- ATR (phospho S428)	Abcam	Cat# ab178407; RRID: AB_2721899
Rabbit anti-NPM/Nucleophosmin	Beyotime	Cat# AG2755; RRID: AB_3698673

**Bacterial and virus strains**		

DH5α Super Competent Cells	Beyotime	D1031S

**Chemicals, peptides, and recombinant proteins**		

KU60019	MedChemExpress	HY-12061; CAS: 925701-46-8
CX5461	Sigma-Aldrich	5.09265; CAS:1138549-36-6
Actinomycin D	MedChemExpress	HY-17559; CAS:50-76-0
Etoposide	MedChemExpress	HY-13629; CAS:33419-42-0

**Critical commercial assays**		

Senescence β-Galactosidase Staining Kit	Beyotime	C0602
BeyoClick™ EdU Cell Proliferation Kit with AF555	Beyotime	C0075S
Mouse IL-1 beta ELISA Kit	ProteinTech	KE10003-96T
Mouse IL-6 ELISA Kit	ProteinTech	KE10007-96T
Comet assay kits	Biogradetech	D-AKE3040-15T
One Step TUNEL Apoptosis Assay Kit	Beyotime	C1089

**Deposited data**		

Acceleration of beta cell aging plays a key role in diabetes and senolysis improves metabolic, functional and cellular outcomes	Joslin Diabetes Center	GEO: GSE121539

**Experimental models: Cell lines**		

HEK293T cell	Abcam	Cat# ab282205, RRID:CVCL_0063
INS-1 cell	SUNNCELL	SNL-332; RRID:CVCL 0352
Beta -TC-6 cell	Cell Bank, Chinese Academy of Sciences	SCSP-5061; RRID:CVCL_0605

**Experimental models: Organisms/strains**		

Mouse:C57BL/6J	The Laboratory Animal Center of Nantong University	N/A

**Oligonucleotides**		

Primers for mouse GAPDH, p16, p21, p53, TIF-IA, 45S and EIF1, and rat 18S, 28S, 45S, Eif1, 18S and 28S, see Table S1	This paper	N/A

**Recombinant DNA**		

Plasmid: pLKO.1-mTIF-IA-shRNA#1	This paper	N/A
Plasmid: pLKO.1-mTIF-IA-shRNA #2	This paper	N/A
Plasmid: pLKO.1 - TRC control	Addgene	#10879

**Software and algorithms**		

GraphPad Prism 8	Dotmatics	http://www.graphpad.com; RRID: SCR_002798
Fiji (ImageJ)	Schindelin et al.^48^	http://fiji.sc; RRID: SCR_002285

### Experimental model and study participant details

#### Animals

Thirty 8-week-old male C57BL/6 mice (weighing 20–22 g) were purchased from the Laboratory Animal Center of Nantong University. Mice were housed in a specific pathogen-free (SPF) facility under controlled conditions (22°C–24°C, humidity maintained) with a 12-h light/dark cycle, and had *ad libitum* access to food and water. All animal procedures were approved by the Animal Ethics Committee of Nantong University (IACUC20220930-1003) and conducted in accordance with the National Institutes of Health guidelines for the care and use of laboratory animals.

#### Cell culture

Mouse β-TC-6 cells were cultured in high-glucose DMEM supplemented with 15% FBS and 1×penicillin-streptomycin solution. Rat INS-1 cells were grown in RPMI 1640 medium supplemented with 15% FBS, 0.05 nM β-mercaptoethanol, and 1× penicillin-streptomycin. Both cell lines were maintained at 37°C with 5% CO_2_ and passaged every three days. Both cell lines have been authenticated by insulin secretion and morphological examination, and confirmed negative mycoplasma contamination using MycoStrip Mycoplasma Detection Kit (InvivoGen, rep-mys-10).

Pancreatic islets were isolated from C57/B6 male mice aged 4–8 weeks. Briefly, C57/B6 male mice were obtained from the Nantong University Laboratory Animal Center were anesthetised with isoflurane. The common bile duct was clamped with a micro forceps, and injected with 1 mg/ml collagenase P intraperitoneally, followed by reaction stop using 3 mL DMEM, 10% FBS. The pancreatic islets were isolated using density gradient method,^49^ and cultured at 37°C with 5% CO_2_ in complete RPMI-1640 media supplemented with 10% FBS, INS-1 cell supplement (10 mM HEPES, 2 mM L-glutamine, 1 mM sodium pyruvate, and 0.05 mM 2-mercaptoethanol), and pen/strep.

### Method details

#### High-fat high-cholesterol (HFHC) diet model

After a one-week acclimation period under SPF conditions, mice were randomly assigned into two groups: one received a standard AIN-93G chow diet, and the other was fed an HFHC diet containing 42% fat and 0.2% cholesterol for 12 weeks. Body weight was recorded weekly. Following the dietary intervention, a glucose tolerance test was conducted, and pancreatic tissues were collected. Specifically, a 1 mm × 1 mm×3 mm section of the pancreatic tail enriched with islets was harvested for subsequent transmission electron microscopy (TEM) analysis.

#### Nucleolar stress induction

CX-5461 and ActD were diluted into complete medium before use. After 24-h cell passaging, cells were exposed to the indicated concentration of nucleolar stress inducers and subjected to Western blot and immunofluorescence experiments post-exposure (72 and 24 h respectively). To ensure reliability, the experiments were repeated three times, with consistent results.

To systematically evaluate the role of ATM kinase in nucleolar stress-induced cellular senescence, this study employed pharmacological intervention using the specific ATM inhibitor KU60019 with experimental groups designed as follows: mock control group (mock treated); inhibitor control group (treated with 3 μM KU60019 alone); nucleolar stress induction group (treated with 200 nM CX-5461 alone); and combined intervention group (co-treated with KU60019 and CX-5461). Notably, in the combined intervention group, to ensure sustained suppression of ATM kinase activity, cells were pre-treated with KU60019 for 30 min prior to CX-5461 addition, allowing sufficient inhibitor absorption and baseline ATM activity blockade. This temporal sequence in experimental design enables precise differentiation between ATM-dependent and ATM-independent regulatory mechanisms in nucleolar stress signaling.

#### Senescence-associated β-Galactosidase (SA-β-gal) staining

SA-β-Gal staining was performed according to the manufacturer’s instructions. Briefly, after the indicated treatments, cells were washed once with PBS and fixed with fixation solution for 15 min at room temperature. Subsequently, cells were incubated with β-Gal staining solution at 37°C overnight. After incubation, the staining solution was removed, and cells were washed with PBS and mounted for analysis using a digital Leica DM5000B microscope.

#### Western blotting analysis

Cells were lysed in radioimmunoprecipitation assay buffer supplemented with protease inhibitor cocktail and 1 mM PMSF on ice for 30 min. Lysates were centrifuged at 12, 000 × g, 4°C for 15 min, and supernatants were quantified using a BCA protein assay. Equal protein amounts were denatured in 2 × SDS buffer (95°C, 5 min), separated by 10% Sodium Dodecyl Sulfate-Polyacrylamide Gel Electrophoresis (SDS-PAGE), and transferred to PVDF membranes. After blocking with 5% skimmed milk/TBST, membranes were incubated overnight with primary antibodies, followed by HRP-conjugated secondary antibodies for 1.5 h at RT. Protein signals were developed using an ECL kit and imaged on a gel scanning system.^50^^,^^51^ Finally, protein quantification was performed using Fiji software.^52^

#### RNA isolation and quantitative real-time PCR (qRT-PCR)

RNA was isolated using TRIzol reagent according to the manufacturer’s protocol. cDNA was reverse transcribed using the RevertAid First Strand cDNA Synthesis kit and subjected to qRT-PCR analysis using Powerup SYBR Green PCR Master Mix on a 7500 Fast real-time PCR system. Primers used for RT-PCR assay were listed in Table S1.

#### 5-Ethynyl-2′-deoxyuridine (EdU) incorporation experiment

The EdU incorporation assay was detected according to the instructions of the EdU Proliferation Kit. Briefly, β-TC-6 cells were evenly seeded into 24-well plates and exposed to CX-54661 and ActD accordingly. Then, EdU incorporation and subsequent fluorescence staining of the cells were carried out in accordance with the instructions. The cell nuclei were stained with DAPI. Finally, the incorporation of EdU was detected under a fluorescence microscope (Leica DM 5000B).

#### Trypan blue

Adherent cells were digested with trypsin to prepare single cell suspensions and then diluted accordingly. The cell suspension was mixed with 0.4% trypan blue solution at a ratio of 9:1 (final concentration was 0.04%). Viable and dead cells were counted within 3 min. Under the microscope, dead cells were stained blue, while viable cells, which refused to be stained, appeared colorless and transparent. Cell viability was calculated, and the viable cell rate (%) = the total number of viable cells/(the total number of viable cells + the total number of dead cells) × 100%.

#### Enzyme-linked immunosorbent assay (ELISA)

Following a 72-h exposure of β-TC-6 cells to CX-5461 and a 24-h exposure to ActD, the Mouse IL-1 beta ELISA Kit and Mouse IL-6 ELISA Kit were employed. The procedures were conducted in accordance with the instructions provided in the kits, and the absorbance was subsequently measured using a microplate reader (Infinite M200 PRO, Tecan, Mannedorf, Switzerland).

#### Immunofluorescence

Cells were fixed with 4% paraformaldehyde, permeabilized with 1% Triton X-100 and blocked with 5% BSA. Thereafter, cell slides were incubated with primary antibodies overnight at 4°C, followed by Alexa Fluor-488-conjugated goat anti-rabbit IgG and DAPI counterstaining. The signal was examined under a Leica confocal microscope (Leica TCS SP8, Leica, Germany).

#### Immunohistochemistry

Tissue sections were deparaffinized with graded ethanol series, followed by endogenous peroxidase blockade with 3% H_2_O_2_ at room temperature for 25 min. After blocking with 3% BSA for 30 min, sections were incubated with primary antibodies overnight at 4°C in a humidified chamber. Subsequently, slides were washed with PBS, incubated with HRP-conjugated secondary antibodies for 50 min, and subjected to signal development using DAB reagents. Reactions were quenched by tap water rinsing. Sections were counterstained with hematoxylin, dehydrated, and mounted for histopathological analysis.

#### Comet assay

The comet assay was conducted using a comet assay kits according to the manufacturer’s instructions. Briefly, β-TC-6 cells exposed to CX-5461 or Etoposide were mixed with melted agarose and lysed using pre-cooled lysis buffer for 30 to 60 min. Next, the slides were treated with alkaline solution, followed by agarose electrophoresis. Slides were dried naturally, stained with PI and examined under a digital fluorescence microscope (TI2-S-HU, Nikon, Tokyo, Japan). The tail moment scores were obtained from at least 50 cells per sample using the OpenComet software.

#### shRNA construction and transduction

shRNA oligos targeting mouse TIF-IA were designed using the Genetic Perturbation Platform (GPP) of Broad institute and subcloned into pLKO.1-TRC vector following standard protocols. Lentiviral particles containing either non-targeting control (NTC) or TIF-IA-targeting shRNAs were packaged by co-tranfecting the shRNA vectors and lentiviral packaging plasmids (pMD2.G and psPAX2) into HEK293T cells using Lipofectamine 2000. For knockdown experiments, β-TC-6 cells at 30% confluency were transduced with the shRNA lentiviruses and selected with 2 μg/mL puromycin for 48 h. Knockdown efficiency was assessed by qRT-RNA analysis, and cells were subsequently examined for senescence-associated phenotypes.

#### TUNEL staining

The TUNEL Staining was conducted using a One-step TUNEL apoptosis detection kit following the manufacturer’s instructions.

### Quantification and statistical analysis

Images were manipulated and analyzed via GraphPad Prism8 and Fiji software. All data were expressed as mean ± SEM. For experiments with technical replicates, the data shown are representative of three independent biological experiments yielding similar results. One-way analysis of variance (ANOVA) was used to compare multiple variables, followed by post hoc comparisons. For datasets with two independent variables, two-way ANOVA with Sidak’s multiple comparisons adjustment was applied. Unpaired two-tailed student’s t-tests were performed for two-group comparisons. *p* < 0.05 was considered statistically significant. Statistical significance was presented as follow: *p*^ns^ > 0.05, *p*^∗^ < 0.05; *p*^∗∗^ < 0.01; *p*^#^ < 0.001.
