# Supplementary material for: Nucleolar stress facilitates islet β cell senescence via hijacking the DNA damage response pathways
Source: iScience. 2025 Sep 4;28(10):113508. doi: 10.1016/j.isci.2025.113508 (PMC12478132; doi:10.1016/j.isci.2025.113508)
Supplement: Document S1. Figures S1–S3 and Table S1 [file mmc1.pdf]

iScience, Volume *28*

Supplemental information

Nucleolar stress facilitates islet β cell senescence via hijacking the DNA damage response pathways

Yaqi Jiao, Weirong Lu, Xiaohua Wang, Wenxing Sun, Baoying Hu, Jianya Zhao, Jinghao Fang, Ying Lu, and Chunhua Wan

Supplemental information

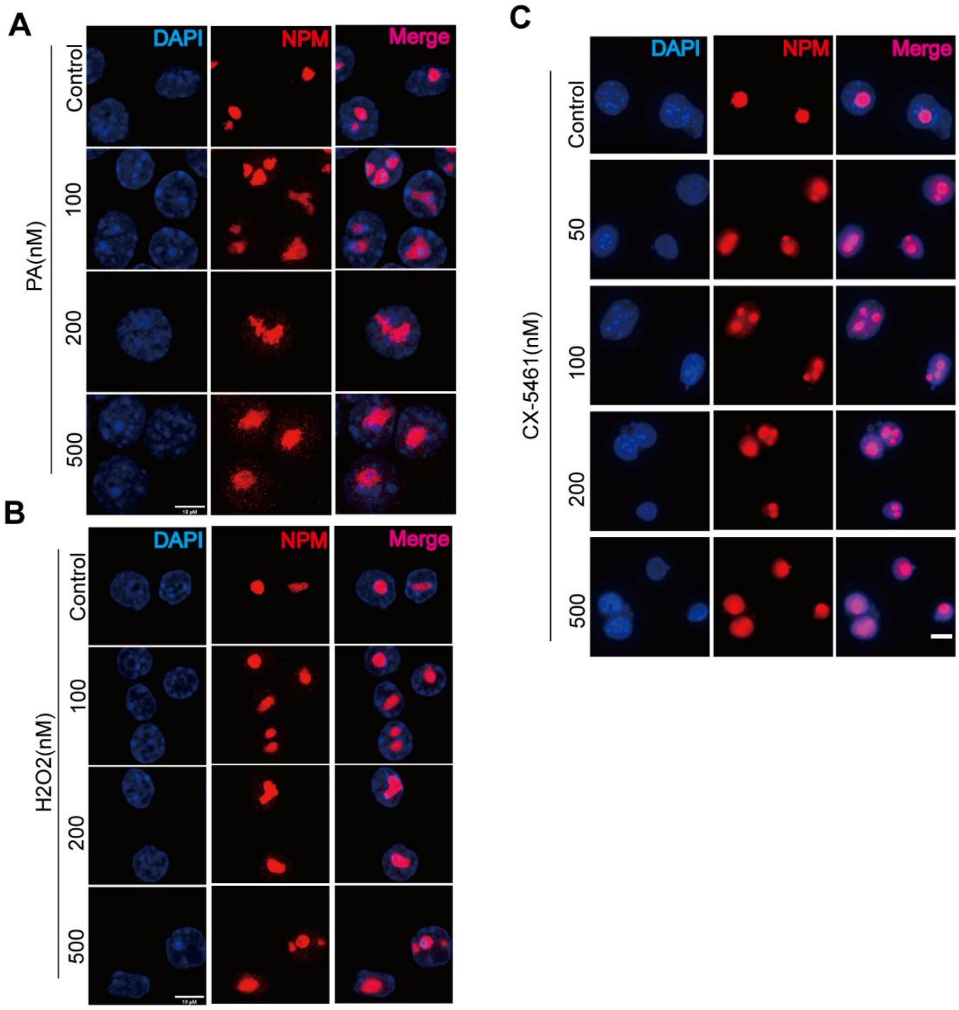

Figure S1. Immunofluorescence analysis of NPM nucleoplasmic translocation under stress conditions.

(A) Immunofluorescence staining of NPM in β-TC-6 cells treated with palmitic acid (PA) for 24 hours (Scale bar, 10μm).

(B) Immunofluorescence staining of NPM in β-TC-6 cells treated with hydrogen peroxide for 24 hours (Scale bar, 10μm).

(C) Immunofluorescence staining of NPM in β-TC-6 cells treated with CX-5461 for 72 hours (Scale bar, 10μm).

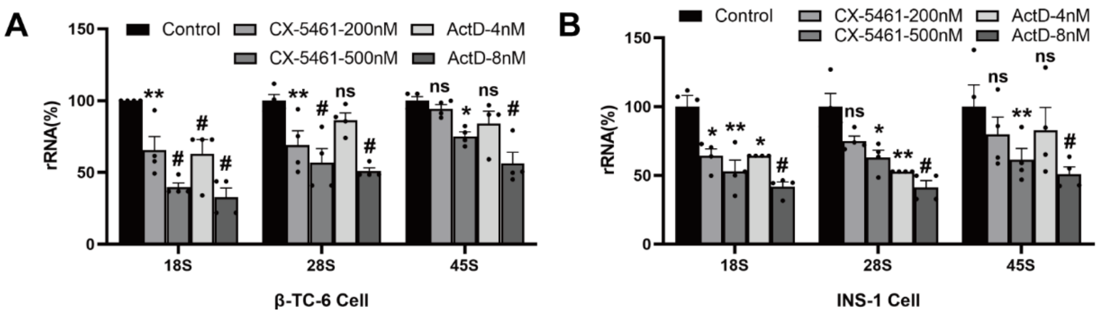

Figure S2. Analysis of rRNA expression following exposure nucleolar stress inducers.

(A) qRT-PCR analysis of 18S, 28S, and 45S rRNA expression levels in β-TC-6 cells treated with CX-5461. Data are mean ± SEM; n=4 independent biological replicates; Dunnett's HSD test compared with the control: $p^{ns} > 0.05$, $p^{*} < 0.05$, $p^{**} < 0.01$, $p^{\#} < 0.001$.

(B) qRT-PCR analysis of 18S, 28S, and 45S rRNA levels in β-TC-6 cells expression levels in treated with ActD. Data are mean ± SEM; n=4; Dunnett's HSD test compared with the control: $p^{ns} > 0.05$, $p^{*} < 0.05$, $p^{**} < 0.01$, $p^{\#} < 0.001$.

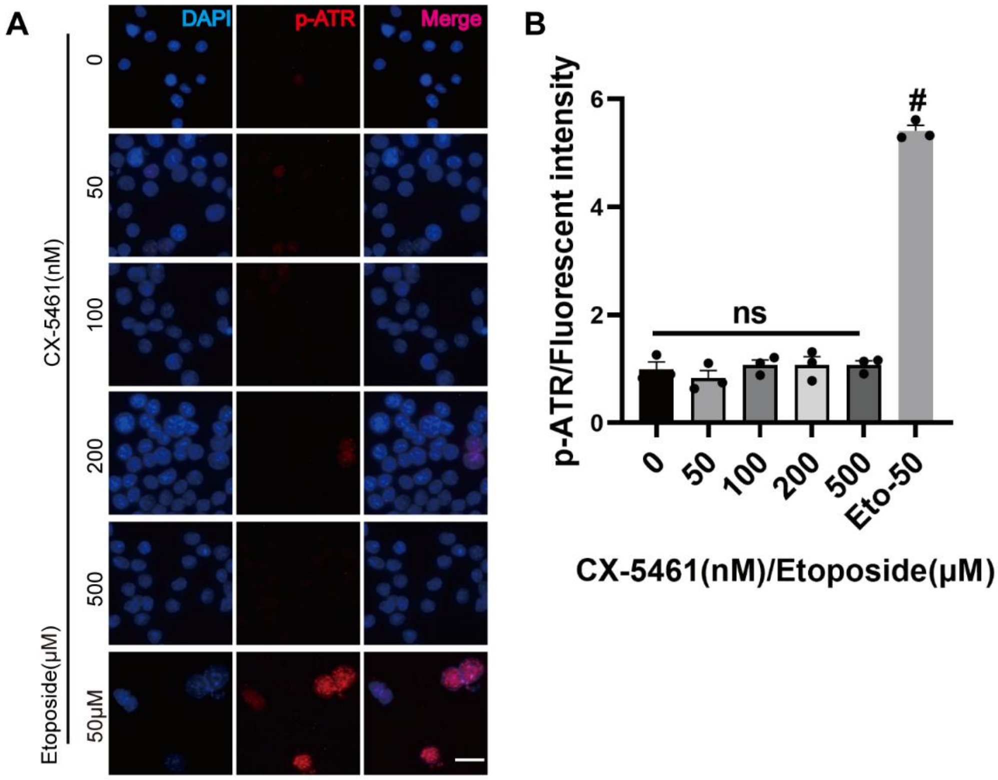

Figure S3. Analysis of phosphorylated-ATR (p-ATR) following CX-5461 exposure.

(A) Immunofluorescence analysis of phosphorylated-ATR (p-ATR) in β-TC-6 cells exposed to CX-5461 for 72 hours (Scale bar, 20μm).

(B) Quantitative analysis of p-ATR fluorescence intensity. Data are mean ± SEM; n=3 independent biological replicates. Dunnett's HSD test compared with the control: $p^{ns} > 0.05$, $p^{\#} < 0.001$.

TableS1: qRT-PCR and shRNA Primers

Primers	Forward primer	Reverse primer
GAPDH(mouse)	GGTCGGTGTGAACGGATTTGG	CCGTGAGTGGGAGTCATACTGGAA
p16(mouse)	CCCAACGCCCCGAACT	GCAGAAGAGAGCTGCTACGTGAA
p19(mouse)	TCTGGAAGAAGTCTGCGTCG	CTTCCAAACATCATGACCTGC
p21(mouse)	GGCAGACCAGCCTGACAGAT'	TTCAGGGTTTTCTCTTGCAGAAG
Rrn3(mouse)	TCAGTTCAGGCCGAATGGC	CGAGCATATCGGAAAGCCCA
18S(rat)	CGCCGCTAGAGGTGAAATTCT	CCAACCTCCGACTTTCGTTCT
28S(rat)	AGTCGGGTTGCTTGGGAATGC	CCCTTACGGTACTTGTTGGCT
45S(rat)	TTAACGGAGAAGGCCGAGG	CGGCGAGAAGGAGGAACACT
Eif1(rat)	AAGGGATCGCTGATGATTACG	GCTGGTCACCCTGTAGCTGA
18S(mouse)	CTGGATACCGCAGCTAGGAA	GAATTTCACCTCTAGCGGCG
28S(mouse)	CGGCGGGAGTAACTATGACT	GCTGTGGTTTCGCTGGATAG
45S(mouse)	GGCTGGGGTTGGAAAGTTTC	CAAGGGCATTCTGAGCATCC
EIF1(mouse)	TGGTACTGTAATTGAGCATCCAG	CCTTAGCCAGCCCAATCTCT
mTIF-IA-shRNA#1	CCGGCCTATTTATCAGATTTGGGAACTCGAGTTCCCAAATCTGATAAATAGGTTTTTG	CCGGCCTATTTATCAGATTTGGGAACTCGAGTTCCCAAATCTGATAAATAGGTTTTTG
mTIF-IA-shRNA#2	CCGGGCAAATTATATTGGGAGCTTTCTCGAGAAAGCTCCCAATATAATTTGCTTTTTG	AATTCAAAAAGCAAATTATATTGGGAGCTTTCTCGAGAAAGCTCCCAATATAATTTGC
